# Native Valve Infective Endocarditis Caused by Dual Infection of Staphylococcus aureus and Pseudomonas monteilii: A Case Report

**DOI:** 10.7759/cureus.97466

**Published:** 2025-11-21

**Authors:** Awais S Butt, Anees S Butt, Nikhil Patel, Sadat Edroos

**Affiliations:** 1 Cardiology, Luton and Dunstable University Hospital, Luton, GBR; 2 Acute Medicine, Luton and Dunstable University Hospital, Luton, GBR; 3 Geriatrics, Luton and Dunstable University Hospital, Luton, GBR

**Keywords:** infective endocarditis, pseudomonas infections, pseudomonas monteilii, staphylococcus aureus endocarditis, transesophageal echocardiography (tee)

## Abstract

Infective endocarditis (IE) is most commonly associated with Gram-positive bacteria such as *Staphylococcus aureus*. Gram-negative causes are less common, and are typically associated with pacemakers, prosthetic valves or intravenous drug use. *Pseudomonas monteilii* is an environmental organism that is rarely reported in human infection, with its presence in sputum often regarded as colonisation or contamination. This case describes a 30-year-old man with no underlying structural heart disease who was found to have a 6-8mm mitral valve vegetation on echocardiography. His blood cultures grew *Staphylococcus aureus, *whilst his sputum cultures grew *Pseudomonas monteilii*. This represents the first documented case of native valve infective endocarditis caused by both *Staphylococcus aureus* and *Pseudomonas monteilii*. This case highlights the diagnostic challenges associated with atypical organism growth and highlights the need to integrate imaging and microbiological findings within the clinical context when uncommon organisms are detected. Ultimately, this rare case broadens the recognised microbiological spectrum of infective endocarditis and supports a high index of suspicion, early imaging, repeat investigation, and multidisciplinary care to achieve good outcomes.

## Introduction

Infective endocarditis (IE), defined as inflammation of the endocardium and the cardiac valves, remains a serious infection with an incidence of 2.6 to 7 cases per 100,000 of the population per year [1,2]. Despite advances in antimicrobial therapy and surgical procedures, mortality remains high [1]. IE is commonly categorised into native valve, prosthetic valve, and intravenous drug use (IVDU)-associated disease. In native valve endocarditis (NVE), major risk factors include rheumatic valvular disease, congenital heart disease (such as patent ductus arteriosus and tetralogy of Fallot), mitral valve prolapse and degenerative heart disease [3].

In terms of pathogenesis, Gram-positive bacteria account for 80% of IE cases, with Staphylococcus aureus (S. aureus) responsible for one-third of IE cases in developed countries [2]. In contrast, gram-negative organisms, namely Pseudomonas, account for only 7% of IE cases and are more commonly associated with pacemakers, prosthetic valves or IVDU [3]. Pseudomonas endocarditis is typically more complex to treat, and when identified, requires dual antibiotic regimes due to the potential for treatment failure with monotherapy [3].

*Pseudomonas monteilii (P. monteilii)* rarely results in clinical disease, with sparse reports of pneumonia, bronchiectasis and device-related infections [4]. Its role in IE has only been described once, in a Spanish study of a patient with a recent prosthetic aortic valve replacement [5]. We report what we believe to be the first case of NVE with dual involvement of *S. aureus* and *P. monteilii* in a young patient with no prior structural heart disease.

## Case presentation

History and physical examination

A 30-year-old male presented to the Emergency Department (ED) with a five-day history of progressively worsening central, non-pleuritic chest pain radiating to his left arm, associated with fever and malaise. He denied intravenous drug use, had multiple tattoos, all performed under sterile conditions, and a history of recent travel to the Philippines (six months prior) and Poland (two weeks prior to admission). His past medical history included a previous episode of septic arthritis and incomplete dental treatment for a symptomatic lower right wisdom tooth. He reported occasional tobacco use, intranasal cocaine use three weeks prior to admission, and an alcohol consumption history of 30 units weekly, with abstinence for 80 days preceding admission.

On examination, his observations showed haemodynamic stability: blood pressure 102/68, heart rate 90, respiratory rate 19, 02 saturations 98% on room air. Chest auscultation demonstrated equal air entry with bi-basal crackles, and the right side of his chest was tender to palpation. There was no audible murmur present. His Glasgow Coma Scale (GCS) was 15/15, his abdomen and calves were both soft and non-tender, and there was no evidence of a rash or any neck stiffness. 

Investigations

ECG demonstrated normal sinus rhythm. Highly sensitive troponin was within normal range. Inflammatory markers were elevated with a CRP of 244mg/L. COVID-19 testing was negative. The chest radiograph was unremarkable. A speculative diagnosis of a community-acquired pneumonia with a low CURB-65 score was made, and he was discharged with oral co-amoxiclav with a review scheduled for the next day. His repeat blood tests on day two indicated a rising CRP (317mg/L), and he was admitted for investigation. 

**Figure 1:**
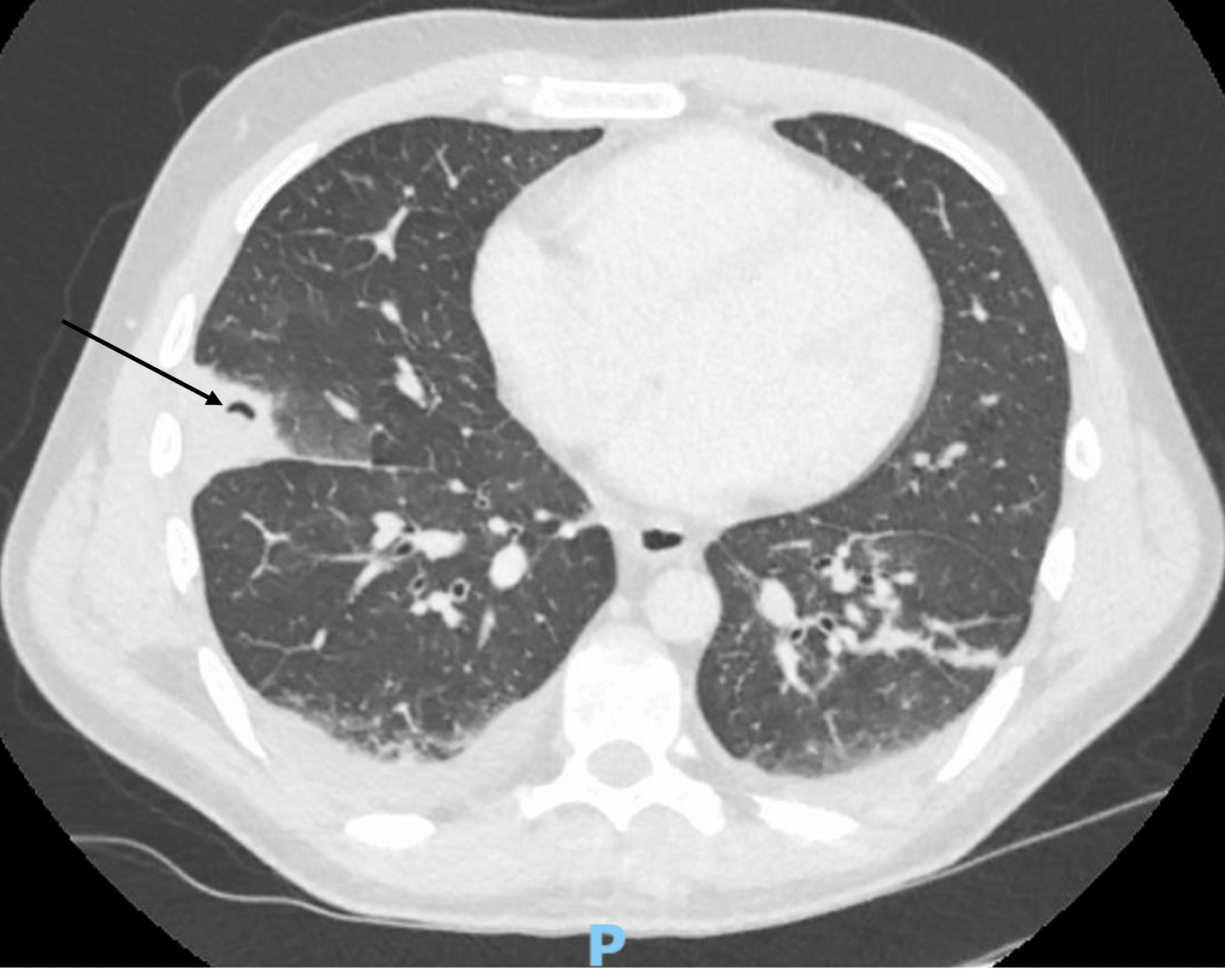
CT of the chest showing a right-sided wedge-shaped consolidation with surrounding cavitation (arrow)

Initial blood cultures grew *S. aureus*, sensitive to flucloxacillin. Other microbiological investigations included a negative urine culture and a sputum culture that isolated *P. monteilii*. Repeat chest radiography on day three demonstrated patchy bilateral consolidation with small effusions. CT chest showed cavitating wedge-shaped consolidation of the right middle lobe, consistent with a septic embolus, with surrounding ground-glass change and bilateral effusions (see Figure 1). Oral and maxillofacial review excluded a dental abscess as the source of infection.

**Figure 2:**
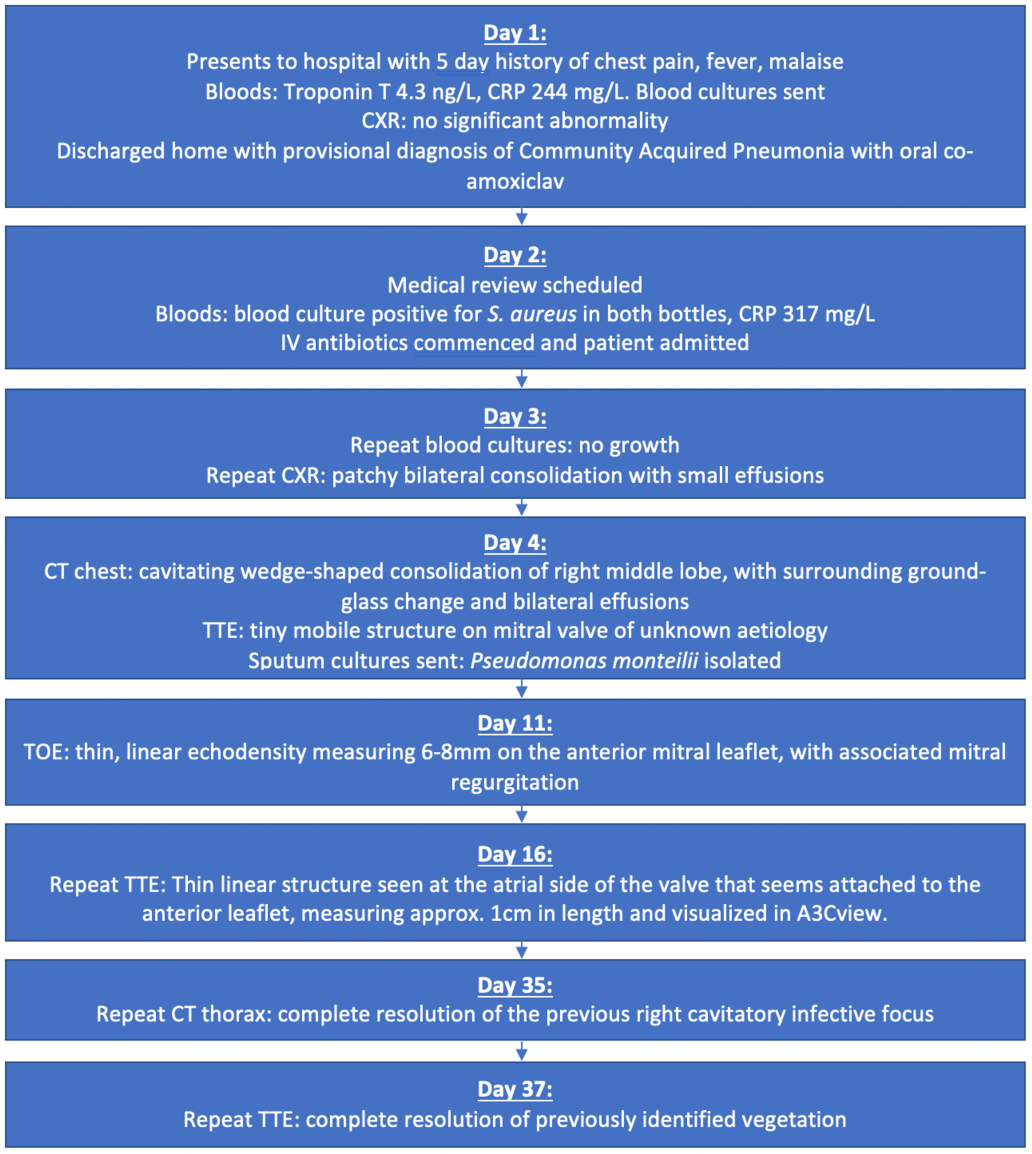
A chronological summary of key investigations and imaging findings CXR = Chest X-Ray, TTE = Transthoracic Echocardiogram, TOE = Transoesophageal Echocardiogram

Transthoracic echocardiography (TTE) identified a subtle mobile structure of the mitral valve. Transoesophageal echocardiography (TOE) confirmed a thin, linear echodensity measuring 6-8mm on the anterior mitral leaflet, consistent with a vegetation, with associated mild mitral regurgitation (see Videos 1, 2). A chronological summary of the key investigations, microbiological results, imaging findings and clinical progression is presented in Figure 2 below.

**Video 1:** Transoesophageal echocardiography showing vegetation on mitral valve leaflet

**Video 2:** Mitral valve regurgitation seen on echocardiography

Treatment

The case was discussed by a multidisciplinary team of cardiothoracic surgeons, cardiologists, and microbiologists. Given the patient’s clinical stability and absence of surgical indications, as per the European Society of Cardiology (ESC) guidelines, a conservative medical management approach was agreed upon [6]. Prior to the availability of blood culture results, the patient received empirical broad-spectrum therapy with intravenous piperacillin-tazobactam. This was subsequently tailored to high-dose intravenous flucloxacillin administered six times daily once blood culture and sensitivity results confirmed *S. aureus*. This was continued for six weeks. Following sputum culture growth of *P. monteilii*, whilst the laboratory did not report formal antibiotic susceptibility results, his case was re-discussed with microbiology specialists who advised a synergistic antimicrobial regimen of linezolid and gentamicin.

Weekly surveillance echocardiograms showed no progression of valvular disease, and the images at day 37 showed resolution of the vegetation. This was not seen on subsequent scans. A repeat CT thorax at day 35 showed complete resolution of the pulmonary cavitation. Repeat blood cultures throughout admission remained negative on antibiotic therapy. The patient remained clinically stable and was discharged following completion of antibiotic treatment, without surgical intervention. 

## Discussion

Rare organisms: *P. monteilii*

We describe a rare co-infection in native valve IE involving *P. monteilii* and* S. aureus*, previously unreported in this context. Whilst *S. aureus* infection is well-documented as the responsible pathogen for 30% of IE cases in developed countries, *P. monteilii* has rarely been reported in human infection [2]. *P. monteilii,* a non-fermenting Gram-negative bacillus isolated from natural environments, is a rare species of *Pseudomonas*, and is often found in healthcare environments on sink surfaces, taps, showers and bedside tables [7]. As previously stated, it infrequently results in clinical disease, with sparse reports of its pathophysiology in human disease.

*P. monteilii *has previously been documented in two cardiac-related diseases. Firstly, it was reported to cause gram-negative sepsis following cardiac monitor insertion [8]. The second, the only other reported case of IE caused by *P. monteilii,* was described in a Spanish study following a recent prosthetic aortic valve replacement [5]. In this Spanish case, the patient was significantly older (76), and imaging demonstrated a vegetation on the prosthetic aortic valve with an aortic pseudo-aneurysm and aortic root abscess. In contrast to our case, this Spanish patient had co-infection with *Acinetobacter nosocomialis* instead of *S. aureus* and underwent surgical management, with a fatal outcome at 44 days [5].

Diagnostic challenges

Although *P. monteilii* was isolated from this patient’s sputum culture, it does not constitute diagnostic evidence of IE as per the ESC guidelines, for two reasons [6]. First, sputum cultures are not recognised as a diagnostic modality, despite their documentation in other case reports, since current guidance focuses on blood cultures and serology [4]. Second, *P. monteilii* is not currently listed as an organism consistent with IE because evidence of its pathogenicity is limited. This, therefore, reinforces ESC guidance for comprehensive microbiological investigation when non-typical organisms are identified and highlights the importance of interpretation of microbiological findings in conjunction with clinical judgement, in order to avoid premature dismissal of organisms labelled as ‘environmental’ or ‘likely contaminants’.

IE remains diagnostically challenging due to its highly variable clinical presentation, from an acute, rapidly-progressive infection, to a chronic disease with no fever or non-specific symptoms [6]. Classical features of IE as per the European Infective Endocarditis Registry (EURO-ENDO) include fever (77.7%), cardiac murmur (64.5%) and congestive heart failure (27.2%) [9]. However, given its varying clinical presentation and the low sensitivity and specificity of clinical signs, the 2023 ESC guidelines advise that IE should be considered in all patients presenting with sepsis or fever of unknown origin, particularly where risk factors are present [6]. This is further supported by the ESC Textbook of Cardiovascular Medicine which reports fever, chills, malaise and night sweats as non-specific symptoms of IE [10]. 

In this case, the patient’s chest pain, fever and malaise initially suggested early-onset pneumonia, and his first chest radiograph was normal. These atypical features resulted in early misclassification of his symptoms and his initial discharge from hospital with oral antibiotics. Thus, this case reinforces the importance of maintaining a high index of suspicion and low threshold for repeat investigation for IE when inflammatory markers are elevated despite negative initial findings.

The ESC guidelines on the modified diagnostic criteria of IE remain central to diagnosis [6]. Our patient fulfilled the criteria on the basis of one major criterion (echocardiographic vegetation) and three minor criteria (fever, a single positive blood culture for *S. aureus* not meeting the major criteria and pulmonary septic emboli). Overall, in this case, there were two positive microbiological findings of note -* S. aureus* evidenced on blood cultures and *P. monteilii *isolated via sputum cultures. It is worth noting that only the initial blood culture taken prior to commencement of broad-spectrum IV therapy with piperacillin-tazobactam tested positive for organism growth. This demonstrates an acknowledged challenge within the ESC diagnostic criteria, whereby subsequent blood cultures may be negative secondary to previous antibiotic administration.

Role of echocardiography and imaging

Diagnosis of IE is largely based on imaging findings, ranging from echocardiography to CT. Echocardiography remains a fundamental imaging modality to identify structural lesions and guide management therapies. The current ESC guidelines recommend TTE as the first-line imaging modality in all suspected cases of IE, with TOE used to confirm findings, particularly in cases of left-sided disease [6]. In this case, TOE confirmed a linear vegetation on the anterior mitral leaflet, consistent with a major diagnostic criterion for IE. In addition, weekly echocardiography, in accordance with the guidelines, was undertaken to monitor the degree of functional valvular damage and assess for complications, confirming stability of the lesion and eventual resolution, supporting the decision for medical management [6].

In this case, CT imaging played a key role in investigating extra-cardiac complications of IE, such as septic emboli, which are reported to occur in up to 25% of patients [6]. CT of the chest and abdomen demonstrated a cavitating pneumonia, most consistent with septic emboli, thus fulfilling one minor criterion in support of a diagnosis of IE. Although pulmonary involvement is more typically associated with right-sided disease, it is also well reported in cases of *S. aureus *IE. This is particularly relevant here, given the patient’s co-infection with both *S. aureus* and *P. monteilii*. The complete radiological resolution demonstrated on repeat imaging highlights the effectiveness of early focused antimicrobial treatment.

Antimicrobial therapy

Throughout this patient’s hospital stay, his antibiotic regimen was regularly reviewed and adjusted under microbiology guidance. Initially, due to his raised inflammatory markers and fever, empirical intravenous piperacillin-tazobactam was initiated. This provided broad Gram-positive and Gram-negative coverage, including activity against both *S. aureus* and *P. monteilii*, which were subsequently identified on microbiological sampling. This is supported by ESC guidance, which recommends empiric treatment should cover both staphylococci and Gram-negative organisms until culture results are available [6]. Once *S. aureus* was confirmed, antimicrobial therapy was rationalised to high-dose intravenous flucloxacillin, the current recommended first-line therapy for methicillin-sensitive *S. aureus* IE [11]. Gentamicin, an aminoglycoside with activity against Gram-negative organisms, was added following isolation of *P. monteilii* on sputum culture to ensure targeted coverage. This approach aligns with published evidence supporting the use of aminoglycosides in *Pseudomonas* infections, demonstrated in both in vivo and in vitro studies [12,13]. In accordance with ESC guidelines for NVE, the patient was treated with prolonged parenteral therapy for six weeks, with negative follow-up cultures and resolution of the identified vegetation [6].

## Conclusions

This case represents the first documented case of native valve IE due to co-infection of both *S. aureus* and *P. monteilii*, expanding the limited evidence of *P. monteilii* as a clinically relevant human pathogen. Previously, *P. monteilii* has only been documented in association with prosthetic valve IE and non-cardiac infections. By detailing native valve involvement, this case expands the microbiological spectrum of IE and demonstrates the importance of interpreting culture results in conjunction with clinical and imaging findings, rather than prematurely dismissing atypical organisms as contaminants.

Although *P. monteilii* is not currently classified as an established IE diagnostic organism, its early isolation alongside *S. aureus *during the acute illness phase, concurrent with severe inflammatory response and radiological features consistent with septic emboli, and resolution only following targeted antimicrobial therapy against both organisms, collectively support that *P. monteilii* contributed to true co-infection rather than colonisation. By the same token, it underlines the diagnostic utility of non-traditional microbiological samples such as sputum, despite their absence from current guidelines.

Overall, this case serves to strengthen awareness that IE may arise from unexpected organisms and highlights the importance of maintaining microbiological vigilance when IE is suspected, particularly when presentations are atypical. This case further emphasises the complexity of diagnosing IE, which often presents atypically. It reinforces several key ESC recommendations: maintaining a high index of suspicion, repeating blood cultures when IE is suspected, undertaking comprehensive imaging early, including TTE, TOE, and CT; and adopting a multidisciplinary-based decision-making approach to management. Importantly, this case illustrates that favourable outcomes can be achieved with conservative management in a select patient population, even in the presence of dual infection, provided evidence-based recommendations are followed.

Ultimately, this case underlines the value of reporting rare cases and emphasises the need for further research and case accumulation to inform future diagnostic criteria and microbial databases.
